# Monitoring Brain Activity with Protein Voltage and Calcium Sensors

**DOI:** 10.1038/srep10212

**Published:** 2015-05-13

**Authors:** Douglas A. Storace, Oliver R. Braubach, Lei Jin, Lawrence B. Cohen, Uhna Sung

**Affiliations:** 1 Department of Cellular and Molecular Physiology, Yale University School of Medicine, 333 Cedar Street, New Haven CT 06520; 2 Center for Functional Connectomics, Korea Institute of Science and Technology, Seoul 136-791, Republic of Korea; 3 NeuroImaging Cluster, Marine Biological Laboratory, Woods Hole, MA 02543

## Abstract

Understanding the roles of different cell types in the behaviors generated by neural circuits requires protein indicators that report neural activity with high spatio-temporal resolution. Genetically encoded fluorescent protein (FP) voltage sensors, which optically report the electrical activity in distinct cell populations, are, in principle, ideal candidates. Here we demonstrate that the FP voltage sensor ArcLight reports odor-evoked electrical activity in the *in vivo* mammalian olfactory bulb in single trials using both wide-field and 2-photon imaging. ArcLight resolved fast odorant-responses in individual glomeruli, and distributed odorant responses across a population of glomeruli. Comparisons between ArcLight and the protein calcium sensors GCaMP3 and GCaMP6f revealed that ArcLight had faster temporal kinetics that more clearly distinguished activity elicited by individual odorant inspirations. In contrast, the signals from both GCaMPs were a saturating integral of activity that returned relatively slowly to the baseline. ArcLight enables optical electrophysiology of mammalian neuronal population activity *in vivo*.

## Introduction

Traditional optical imaging techniques using intrinsic signals^1^,^2^ or organic dyes for measuring voltage^3^ and calcium^4^,^5^, are limited in their ability to distinguish the cell types contributing to the signal, except in special cases^6^,^7^. In contrast, protein reporters of neural activity can be targeted to genetically distinct cell types. ArcLight is a protein voltage sensor that can detect single action potentials in cultured mammalian neurons^8^, and *in vivo* in *Caenorhabditis elegans*^9^ and *Drosophila*^10^. These results make ArcLight a strong candidate for *in vivo* functional imaging in mammalian preparations.

However, measurements made in one type of preparation have not necessarily translated to good performance in another. Early protein voltage sensors^11^ resulted in poor expression in mammalian cells^12^, and other sensors that worked well in mammalian cells *in vitro*^13^,^14^ suffered from poor *in vivo* performance^15^,^16^,^17^.

A voltage sensor with a good signal-to-noise ratio could facilitate understanding the function of the mammalian olfactory bulb. Olfactory sensory neuron axons converge in regions of neuropil called glomeruli, forming connections with the apical dendrites of mitral and tufted cells, whose axons project out of the bulb. The transformation accomplished by the olfactory bulb is currently unclear. The networks of bulb interneurons surrounding each glomerulus^18^, the inter-glomerular processing via mitral and tufted cell lateral dendrites, and the feedback projections from cortical areas^19^ must all contribute to this transformation.

Calcium signals have often been used as a surrogate for measuring action potential activity for glomerular measurements^7^,^20^. However, calcium concentration changes are slower than voltage signals, aren’t always present in response to voltage changes, and calcium signals in mitral cells are elicited by subthreshold activity and thus calcium signals do not provide unambiguous information about mitral cell action potential activity^21^. Furthermore, calcium signals will be slowed by the binding kinetics of calcium with the sensor and the slowing will depend on expression levels^22^. A fast voltage sensor would provide a more direct measurement, as recent studies demonstrated that behaviorally relevant information is obtained very rapidly in rodents, in a single sniff^23^,^24^.

The present study used ArcLight to record the population signals that largely came from mitral and tufted cell dendritic tufts in each glomerulus. The ArcLight voltage signals were compared with those from the protein calcium sensors GCaMP3^25^ and GCaMP6f 26 using paired recordings from opposite olfactory bulb hemispheres. ArcLight odor-evoked responses could be detected in the glomerular layer in single trials using wide-field and 2-photon imaging, and simultaneous wide-field measurements from opposite bulbs with ArcLight and GCaMP3 or GCaMP6f revealed that ArcLight’s temporal kinetics were substantially faster than both GCaMPs.

## Results

### Injecting AAV vectors; expression patterns

**Figure 1:**
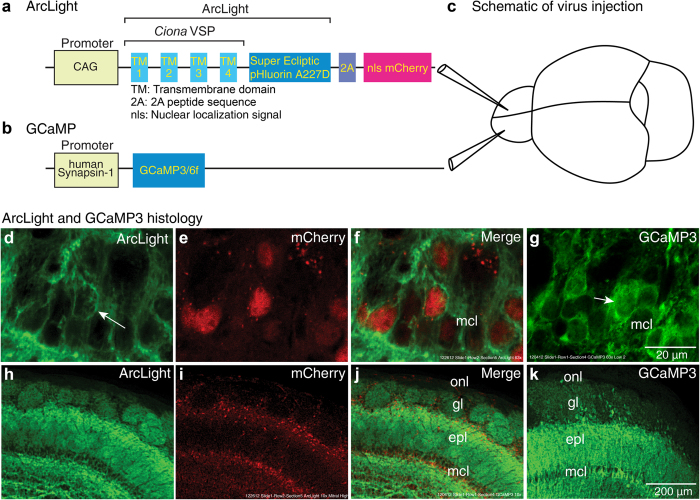
Schematic of constructs, virus injections, and results of histological examination of the fluorescence of ArcLight and GCaMP3 in injected olfactory bulb hemispheres. (**a-b**) Constructs for (**a**) ArcLight and (**b**) GCaMP3/6f AAV1 vectors. The mCherry in the ArcLight construct includes a nuclear localization sequence to facilitate identification of transduced neurons. (**c**) Each AAV vector was injected into an olfactory bulb hemisphere at least 10 days prior to imaging. (**d**) High magnification confocal photomicrographs of ArcLight demonstrate membrane localization (arrow). (**d**) ArcLight, (**e**) mCherry and (**f**) merge. (**g**) High magnification photomicrograph of GCaMP3 fluorescence. The GCaMP3 fluorescence seems to fill the cytoplasm (arrow). (**h-k**) Low magnification confocal photomicrographs demonstrate expression patterns of the ArcLight (**h-j**) and GCaMP3 AAV vectors (**k**). Both vectors appeared to be selective for mitral/tufted cells in the outer layers of the bulb. Scale bars in g and k apply to d-g and h-k, respectively. onl, olfactory nerve layer; gl, glomerular layer; epl, external plexiform layer; mcl, mitral cell layer.

The mouse olfactory bulb responds to different odorants with distinct spatial and temporal patterns of glomerular activation^7^,^27^,^28^. We tested whether ArcLight can be used as an *in vivo* reporter of the glomerular output via mitral/tufted cells, and compared it to the calcium sensors GCaMP3 and GCaMP6f. Adeno-associated virus (AAV1) vectors carrying genes for ArcLight and GCaMP3 or GCaMP6f (Fig. 1a-b) were injected into separate bulb hemispheres between 10 and 50 days prior to optical measurements (Fig. 1c). Injections were targeted towards the rostral part of the bulb but expression tended to be widespread in the injected hemisphere.

Experiments in cultured neurons demonstrated that ArcLight traffics to the plasma membrane^8^. We developed an AAV1 ArcLight vector with the 2A peptide followed by mCherry with a nuclear localization sequence (Fig. 1a) which facilitates identification of transduced neurons. Expression of the ArcLight and the calcium sensors were histologically examined based on the sensor fluorescence. When viewed at high magnification, ArcLight often appeared targeted to neuronal membranes (Fig. 1df, arrow in d), while GCaMP3 expressing neurons mainly had cytoplasmic fluorescence (Fig. 1g, arrow).

At lower magnification, ArcLight (Fig. 1h-j) and GCaMP3 (Fig. 1k) vectors appeared to target similar populations of neurons in the external layers of the bulb. Both were largely selective for mitral and tufted neurons (Fig. 1, compare j, k), although an occasional juxtaglomerular neuron also appeared to be labeled. We presume that the labeled glomeruli are the result of dendritic expression in the primary dendritic tufts of the mitral/tufted neurons.

### Imaging individual glomerular output with ArcLight using wide-field epifluorescence or 2-photon microscopy

**Figure 2 f2:**
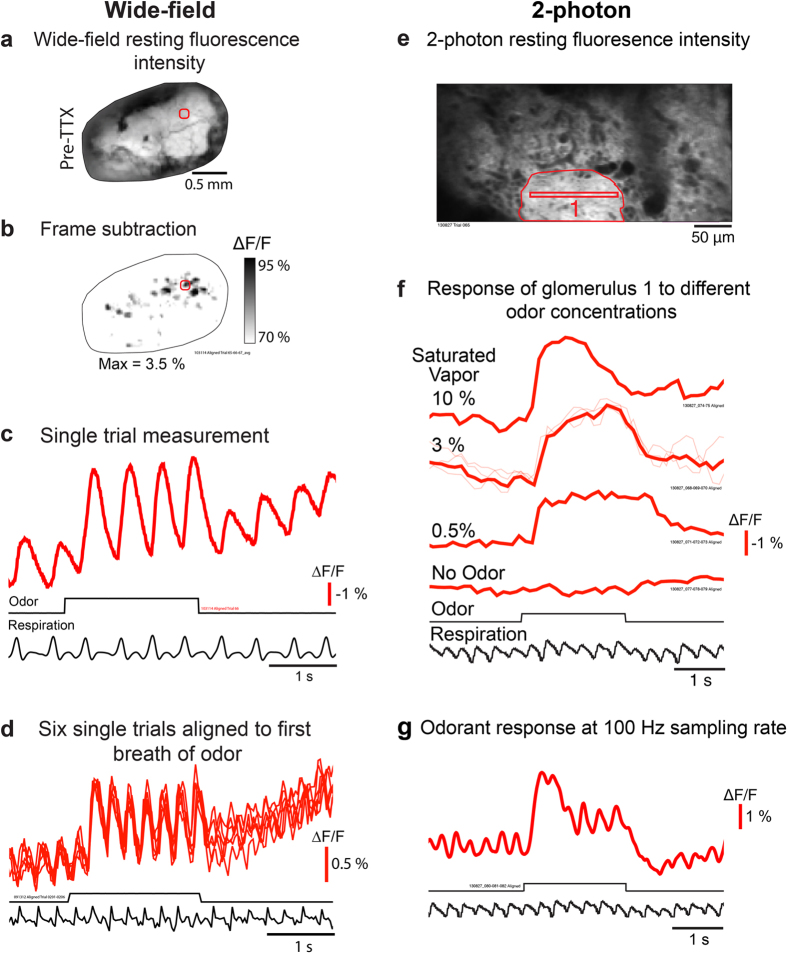
Odor-evoked ArcLight signals in single trials using wide-field and 2-photon microscopy. (**a-d**) Wide-field epifluorescence microscopy. (**a**) Wide-field resting fluorescence intensity. (**b**) Glomerular patterns of activation after odor presentation. (**c**) Odor-evoked optical measurement from the region of interest marked with a red circle in a-b. The frame subtraction map in b is from an average of 3 respiration aligned trials with no spatial filtering. The optical measurement in c is from an unfiltered single trial (the respiration trace is filtered at 4 Hz). (**d**) Six unfiltered single trials aligned to the first sniff of odorant. The respiration trace is from one of the single trials. (**e-g**) 2-photon scanning microscopy. (**e**) 2-photon resting fluorescence intensity from the glomerular layer in the rostral bulb. (**f**) Optical measurements from glomerulus 1 in panel e in response to ethyl tiglate at 3 odor concentrations and an air alone presentation. The optical traces in f are averages of 2-3 respiration aligned trials (thick lines); single trials are shown as thin lines for the 3% saturated vapor condition. Measurements were recorded at a sampling frequency of 8 Hz, and the traces were Gaussian low-pass filtered at 2 Hz. (**g**) Increasing the 2-photon sampling rate improved the signal-to-noise ratio and revealed respiration coupled ArcLight signals. The optical signal in panel g is from the smaller imaging area in panel e (512 × 16 pixels, thin red box in glomerulus 1) and is from an average of 3 respiration aligned trials. The optical traces in g were recorded at a sampling frequency of 100 Hz, and were Gaussian low-pass filtered at 4 Hz. The respiration traces in panels f and g are from single trials. The data in a-c, d, and e-g are from three different preparations.

We first examined whether ArcLight can be used to measure *in vivo* odor-evoked responses from the glomerular tufts of mitral/tufted cells using epifluorescence and 2-photon scanning microscopy. Injections of the ArcLight AAV (Fig. 1a,c) generally resulted in relatively uniform fluorescence across the dorsal surface (Fig. 2a). Odorant presentation evoked glomerular-sized changes in ArcLight fluorescence (Fig. 2b) that were relatively large and fast (Fig. 2c-d,f). ArcLight signals representing depolarizations are shown as upward signals^8^. The optical signals recorded using wide-field epifluorescence (at 125 Hz) were clearly coupled to respiration (Fig. 2c-d). The 2-photon measurements recorded at 8 Hz were not (Fig. 2f), although increasing the 2-photon sampling rate to 100 Hz by reducing the number of scan lines improved the signal-to-noise and resulted in signals that were coupled to respiration (Fig. 2g). Higher concentrations of saturated vapor generally resulted in larger amplitude optical signals (Fig. 2f).

Bath application of TTX to the dorsal surface of the bulb eliminated the response to odorants. TTX significantly reduced the power of the optical signals at the respiration frequency before (74% ± 4 percent decrease, 1-sample t-test: t(3) = −17.4, p < 0.001) and during the odorant presentation (87% ± 2.2 percent decrease, 1-sample t-test: t(3) = −39.2, p < 0.001; 4 preparations). The ArcLight respiration coupled signals during the odorant presentation, and the smaller respiration coupling before and after the odorant presentation are both consistent with electrode recordings from individual mitral/tufted neurons showing that action potential frequency is highly modulated by respiratory activity both in the presence and absence of odor^23^,^29^,^30^.

**Figure 3 f3:**
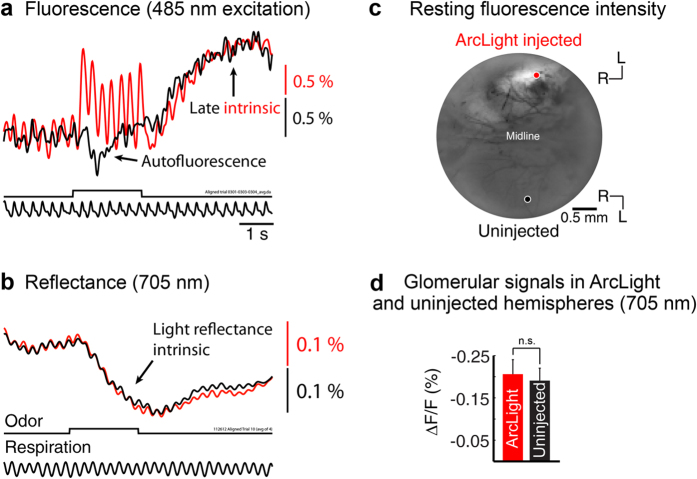
Time course of signals from ArcLight and uninjected hemispheres reveal that ArcLight signals are only present in the injected hemisphere, and the presence of ArcLight does not adversely affect intrinsic signals. (**a**) Measurements from injected (red trace) and uninjected hemispheres (black trace) using 485 nm excitation light and 530 long pass emission. The presumed slow intrinsic light scattering signal (slow upward) is present in both hemispheres, although the flavoprotein autofluorescence signal^52^ is only clearly seen in the uninjected hemisphere. The control trace is taken from an average of three respiration aligned trials. The odor and respiration traces are from a single trial. The traces are low-pass filtered at 4 Hz and inverted. (**b**) Comparison of intrinsic light reflectance signals measured using 705 nm light from the same locations in panel a. Traces are from an average of 4 respiration aligned trials, are low pass filtered at 2 Hz, and are shown in their original orientation. (**c**) Resting fluorescence intensity image of ArcLight injected (top) and uninjected (bottom) olfactory bulb hemispheres measured using 485 nm incident light. Overlaid kernels indicate the locations used for the traces shown in a and b. (**d**) Population statistics comparing glomerular responses measured with intrinsic light reflectance signals at 705 nm in ArcLight injected and uninjected hemispheres. The population comparison is non-significant (p > 0.05 using a 2-sample t-test). Data included in d are from averages of 2-6 trials.

The ArcLight recordings were consistent across trials (Fig. 2d), suggesting that phototoxicity and photobleaching were limited. The ArcLight recordings have a late slow intensity increase that we presume results from interference from an intrinsic optical signal, which has the same direction and a similar slow time course^2^,^31^. Paired recordings from ArcLight injected and uninjected hemispheres had comparable slow changes when measured with the filter set used for the ArcLight fluorescence measurements (Fig. 3a). A relatively small autofluorescence signal in the opposite direction can be seen in the uninjected bulb (Fig. 3a). This signal is not obvious in the ArcLight hemisphere because the relatively dim intrinsic fluorescence is overshadowed by the much brighter ArcLight fluorescence. In addition, intrinsic reflectance signals measured using 705 nm light were comparable in both injected and uninjected bulbs (Fig. 3b,d), suggesting that pharmacological effects of ArcLight was minimal. Similar results were obtained with GCaMP3 (not shown).

### Comparisons of odorant-evoked voltage and calcium signals

**Figure 4 f4:**
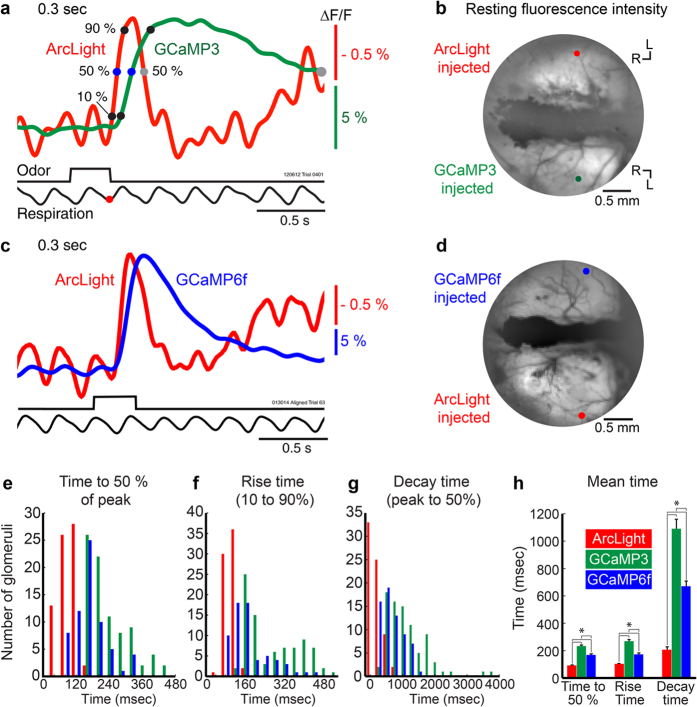
ArcLight and GCaMP responses to a single inhalation of odorant. (**a, c**) Simultaneous measurements made of ArcLight and GCaMP3 (**a**) and ArcLight and GCaMP6f (**c**) injected hemispheres. Traces are from single trials low-pass filtered at 4 Hz. (**b, d**) Resting fluorescence intensity for the preparations in a and c with recording locations indicated by colored circles. (**e-h**) Quantitative comparison of temporal properties of ArcLight, GCaMP3 and GCaMP6f signals from all identified glomerular sized peaks of activation. The first negative peak of the respiration signal following the onset of the odorant was identified for each trial, and was defined as the start time (red circle in a). Selected time points for analysis are indicated as circles on the traces in a. (**e-g**) Histograms showing time to reach 50% of the peak (**e**), the ris**e** time (**f** 10% to 90%), and the time for the peak to decay to 50% (**g**) for ArcLight (red), GCaMP3 (green) and GCaMP6f (blue). (**h**) Bar graphs showing mean (± s.e.m.) for the quantifications in e-g. Asterisks indicate significance at p < 0.001. R, rostral, L, lateral.

Calcium signals are frequently used as a surrogate for action potential measurements *in vivo*. We compared GCaMP signals to those using ArcLight in measurements made simultaneously in opposite hemispheres. Odorants presented for 0.3 s often coincided with a single inhalation, which resulted in ArcLight and GCaMP3 (Fig. 4a-b, red versus green trace) or ArcLight and GCaMP6f (Fig. 4c-d, red versus blue trace) fluorescence signals that were easily detectable in single trials. Increases in calcium from the GCaMPs are shown as upward signals.

The ArcLight and GCaMP signals had distinctly different amplitudes and time courses (Fig. 4a,c). ArcLight had a much smaller fractional fluorescence change (ΔF/F) than either GCaMP. Single inhalations of ethyl tiglate or isoamyl acetate (10% of saturated vapor) evoked ArcLight, GCaMP3 and GCaMP6f signal sizes (ΔF/F) of 1.2% ± 0.05 (6 preparations, 69 glomeruli, range: 0.31-2.6%), 8.9% ± 0.5 (5 preparations, 84 glomeruli, range: 1.3-18.5%) and 24.8% ± 1.3 (4 preparations, 65 glomeruli, range: 7.1-48.1%) respectively. However, ArcLight had a faster onset, rise time, and decay. Quantitative comparisons of the time course of ArcLight (red), GCaMP3 (green) and GCaMP6f (blue) responses to single odorant inhalations were made from glomerular sized peaks of activation across the bulb (Fig. 4e-h). ArcLight signals reach 50% of their maximum more quickly (Fig. 4e,h, F(2, 215) = 112.1, p < 0.0001), have a shorter rise time (Fig. 4f,h, F(2, 215) = 78.23, p < 0.0001), and return to 50% of the baseline faster (Fig. 4g,h, F(2, 214) = 76.26, p < 0.0001) than GCaMP3 and CGaMP6f signals (one-way ANOVAs were used for all comparisons, Tukey post-hoc comparisons confirmed that ArcLight was fastest, followed by GCaMP6f and GCaMP3). Samples for ArcLight, GCaMP3 and GCaMP6f in e-h include 69 (6 preparations), 84 (5 preparations) and 65 (4 preparations) glomeruli, respectively.

**Figure 5 f5:**
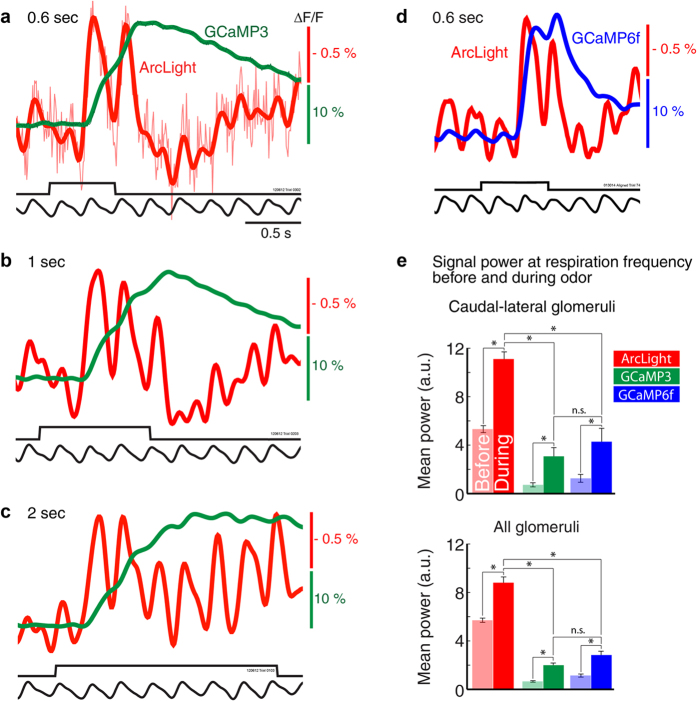
ArcLight, but not GCaMP optical signals have sufficiently fast temporal dynamics to clearly distinguish the neural activity resulting from individual odorant inhalations. (**a-c**) Simultaneous measurements of ArcLight (red) and GCaMP3 (green) signals in response to odorant presented for 0.6 s, 1 s and 2 s (from the same preparation and bulb location as Fig. 4a). (**d**) ArcLight (red) and GCaMP6f (blue) measurements in response to odorant presented for 0.6 s (from the same preparation and bulb position location as Fig. 4c). Longer odorant presentations resulted in breath coupled responses that were very obvious for ArcLight, but relatively small for both GCaMPs. Traces in a-d are from single trials low-pass filtered at 4 Hz. An unfiltered trace is included in a. (**e**) All three sensors had significant increases in power at the respiratory frequency during the odorant presentation (compare light and dark bars). ArcLight responses have significantly higher power (a.u., arbitrary units) than either GCaMP). The analysis in the caudal-lateral bulb used 3 glomeruli from each preparation that were located caudal-laterally, and were also strongly breath coupled. Asterisks indicate significance at p < 0.001.

We increased the length of the stimulus presentation from 0.3 s, to 0.6, 1, and 2 s, which increased the number of odorant inhalations (Fig. 5a-d). Regardless of the odorant duration, ArcLight recordings from the caudal-lateral region of the bulb were strongly coupled to respiration, where each inhalation of the odorant was immediately followed by a peak in the ArcLight signal, and a nearly complete return toward the baseline during subsequent exhalations (Fig. 5a-d,2c,3a, red traces). Inhalation coupled responses were detectable with GCaMP3 (Fig. 5a-c, green traces) and GCaMP6f (Fig. 5d, blue trace), but these were much less prominent. All three sensors had power at the respiration frequency prior to odorant presentation, and all three sensors had a significant increase in power at the respiration frequency during the odor presentation (repeated measures ANOVA F(1, 268) = 236.7, p < 0.0001) (Fig. 5e, compare light vs dark bars). However, a comparison of the three sensors normalized to the peak signal amplitude revealed that ArcLight has significantly more power at the respiration frequency than both GCaMPs before (one-way ANOVA F(2, 268) = 846.3, p < 0.0001) and during odorant presentation (F(2, 268) = 190.2, p < 0.0001) (Tukey post hoc comparisons confirmed ArcLight was largest) (Fig. 5e). Samples for ArcLight, GCaMP3 and GCaMP6f include 73 (6 preparations), 117 (6 preparations) and 81 (5 preparations) glomeruli, respectively.

ArcLight always reached its peak response within a single inhalation of odorant (Fig. 5a-d, *red traces*). In contrast, GCaMP3 was slower and tended to take up to 3 sniffs to reach its peak response for a given odorant concentration (Fig. 5a-c, green traces). GCaMP6f was more responsive than GCaMP3, and reached 80% (± 0.07) of its peak after the first sniff (Fig. 5d, blue trace) at all tested odor concentrations (not shown). When normalized to their peak signal amplitude, GCaMP6f signals were significantly larger than those of GCaMP3 for the first two sniffs (paired t-tests, Sniff1: t(7) = −8, p < 0.001, Sniff2: t(7) = −7.75, p < 0.001, Sniff3: t(7) = 0.39, p = 0.70).

**Figure 6:**
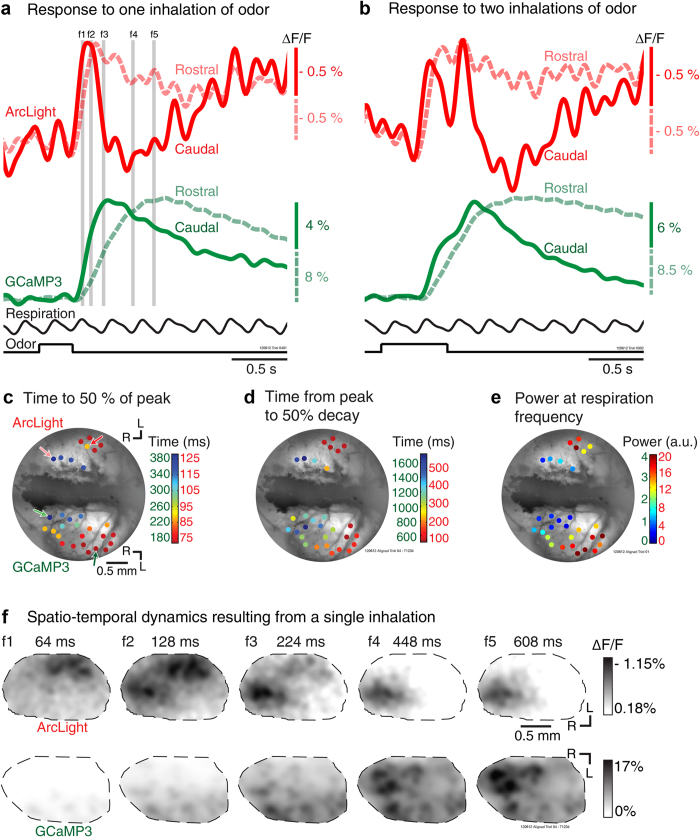
Wide-field microscopy: spatial organization of glomerular temporal heterogeneity. (**a**) ArcLight (red) and GCaMP3 (green) fluorescence traces measured from caudal (solid line) and rostral (dashed line) locations in response to one (**a**) or two (**b**) inspirations of an odorant. Glomeruli in the caudal-lateral bulb responded faster, and had responses that were more tightly coupled to respiration. The traces in a and b are single trials filtered at 4 Hz. (**c, d, e**) Resting fluorescence intensity images with an overlay of activated glomerular regions of interest (colored circles) color coded to represent the time to reach 50% of the peak signal (**c**), the time for the peak signal to decay 50% (**d**), or the power at the respiratory frequency (**e**). The four arrows in C indicate the location of the regions of interest selected for the traces in a and b. (**f**) Frame subtractions of ArcLight and GCaMP3 recordings (average of 4 trials) taken at the time points indicated by the vertical gray bars in a.

Although ArcLight reports neuronal population activity faster than GCaMP3 and GCaMP6f, all three sensors had a large spread of temporal responses across a population of glomeruli (Fig. 4e-g). We examined whether temporal responsiveness was related to the dorsal position on the bulb. Both the voltage and the calcium measurements from rostral-medial glomeruli had slower onsets and a slower return to baseline than those located in the caudal-lateral bulb (Fig. 6a-b, dashed vs solid traces). The time to reach 50% of the peak (Fig. 6c), time for 50% decay (Fig. 6d), or the power at the breathing frequency (Fig. 6e) as a function of bulb position all demonstrated that these temporal properties shift in the caudal-lateral to rostral-medial direction. This functional topography was evident in all five tested preparations and are consistent with previous reports^20^,^32^. Figure 6f shows frame subtractions of signals evoked by a single inhalation illustrating the changing patterns of activation at the times indicated by the gray bars in Fig. 6a. ArcLight signals in faster responding glomeruli in the caudal-lateral region were briefer (Fig. 6f1-f4, [Fig f2], [Fig f3], [Fig f4]), while the signals in slower glomeruli in the rostral-medial bulb were longer lasting (Fig. 6f2-f5, [Fig f3], [Fig f4], [Fig f5]). GCaMP3 signals were slower in onset and offset.

### Comparison of ArcLight and GCaMP sensor response speed

The differences in time course and respiration coupling that we found between ArcLight and the GCaMPs are likely due to a combination of differences in the time course of the cellular voltage and calcium changes as well as the response speeds of the sensors. To further understand these differences, we compared the temporal kinetics of the ArcLight response to changes in membrane potential to published stopped flow data on GCaMP3 and GCaMP6f responses to changes in calcium in solution^26^,^33^,^34^ (D. Kim, L. Looger, S Wang, personal communication).

**Figure 7:**
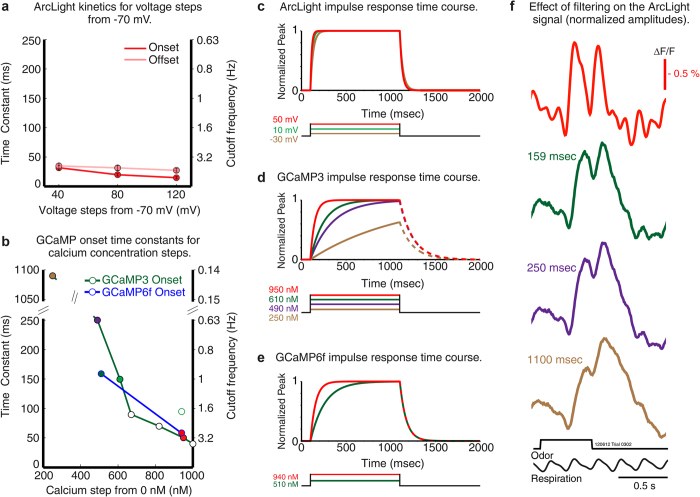
Comparison of sensor kinetic properties. Experimentally measured ArcLight values for different depolarizing steps are compared with published and personally communicated values for GCaMP3 and GCaMP6f. GCaMP3 and GCaMP6f are considerably slower in response to calcium changes than ArcLight is to membrane potential changes. (**a**) ArcLight onset (red) and offset (pink) time constants in response to different depolarizing steps from −70 mV in HEK293 cells. (**b**) GCaMP3 (green line), and GCaMP6f (blue line) onset time constants in response to different calcium steps^26^,^33^,^34^ (D. Kim, L. Looger, S Wang, personal communication). The GCaMP3 onset kinetics (green line) are the result of steps from 0 nM^33^. The green outlined GCaMP3 data point at 940 nM, and both GCaMP6f data points are in response to steps from 100 nM (D. Kim, L. Looger, S Wang, personal communication). (**c, d, e**) Normalized impulse responses for ArcLight (**c**), GCaMP3 (**d**), and GCaMP6f (**e**) to physiologically relevant st**e**ps of voltage and calcium (the selected GCaMP3 steps are indicated by colored data points in b). The GCaMP offset rates in d and e are shown as dashed lines to indicate the possibility that it could be longer for decreases from lower calcium concentrations. (**f**) ArcLight signals in response to two breaths low-pass filtered using time constants equivalent to those of GCaMP3 and GCaMP6f for steps of calcium.

ArcLight had onset and offset time constants of 20 and 30 msec, respectively for 80 mV depolarizing steps in HEK293 cells (Fig. 7a). GCaMP3 has onset time constants of 1100 and 250 msec for calcium steps to 250 nM and 490 nM (Fig. 7b, green line, time constants of 1100 and 250 msec are equivalent to cut-off frequencies of 0.14 Hz and 0.7 Hz)^33^. GCaMP6f is about 50% faster for calcium concentration steps to ~ 510 nM and ~ 940 nM (Fig. 7b, blue line) (D. Kim, L. Looger, S Wang, personal communication). Fig. 7c-e compares the temporal responses for ArcLight (c), GCaMP3 (d) and GCaMP6f (e) for selected steps of voltage and calcium. The decay constants for GCaMP3 and GCaMP6f are reported to be ~150 msec^34^ and 71 msec (D. Kim, L. Looger, S Wang, personal communication), respectively, when starting at a high calcium concentration. The concentration dependence of the decay times has not been reported. ArcLight has faster kinetics than either GCaMP.

Thus, ArcLight’s better ability to detect individual inspiration responses may be in part due to its faster temporal kinetics. To simulate the effect that slower temporal kinetics would have on the ArcLight signal, we determined the time course of the ArcLight signal in response to two breaths when the signal is filtered with a simple RC low-pass filter with time constants of 159, 250 and 1100 msec (equivalent to the on time constants of GCaMP3 for calcium steps of 250 nM, 490 nM and 610 nM) (Fig. 7f). Filtering with a 1100 msec time constant resulted in a onset signal that is similar to the GCaMP3 response onset (Figs 5, 6, green traces), with an increased response amplitude with the second breath. However, none of the filters resulted in a greatly prolonged recovery of the signal like that seen with the GCaMPs (Figs 4, 5, 6). Thus, the GCaMP onset response kinetics appears to have an impact on one, but not all, aspects of the calcium measurement.

## Discussion

We report the *in vivo* response of the genetically encoded FP voltage sensor, ArcLight^8^, in the mammalian brain. Injections of an AAV1 vector resulted in widespread labeling of mitral and tufted neurons. This ArcLight expression made it possible to record odorant-evoked optical signals in single trials *in vivo* using both conventional wide-field optics and 2-photon imaging. Wide-field imaging was used to directly compare ArcLight measurements with those of the genetically encoded calcium reporters GCaMP3 and GCaMP6f 25,26. ArcLight signals had substantially faster temporal dynamics that easily visualized respiratory coupled activity.

The ArcLight signal likely reflects the population average of all the cells and processes expressing the sensor in the glomerular region of interest. Electrode measurements from individual mitral and tufted cells reveal that they produce action potentials that are tightly linked to the respiration cycle, and each cell can be tuned to a different phase of the sniff^23^,^30^,^35^. Thus, our ArcLight measurements appear to reflect the respiration driven envelope of this spiking activity^36^.

The odorant evoked *in vivo* response time course of ArcLight and the GCaMPs are dependent on the kinetics of the voltage and calcium changes they are designed to detect, as well as intrinsic properties of the protein sensors which include sensor expression levels and kinetics^22^. ArcLight and the GCaMPs have onset and decay kinetics that delay their responses to voltage and calcium (Fig. 7a-b). The onset and decay kinetics of the GCaMP sensor responses to step changes in calcium result from the binding and unbinding rates of calcium. The mechanism by which ArcLight works is not known; it has been hypothesized to result from a movement of the S4 trans-membrane segment of the voltage sensitive domain of the *Ciona* protein that is transmitted to the attached super ecliptic pHluorin (A227D) chromophore^8^.

ArcLight’s onset and offset kinetics are faster than those of GCaMP3 and GCaMP6f over the physiological range of voltage and calcium concentrations (Fig. 7a-b)^26^,^34^(D. Kim, L. Looger, S Wang, personal communication). The onset kinetics of the GCaMPs also depend on the calcium concentration (Fig. 7b, d, e). While their offset rates aren’t reported as a function of the starting calcium concentration^26^,^34^, it is plausible that the slow onset rates at lower calcium concentrations could be due to a high affinity calcium binding site with a slow off rate. Thus the offset time constants starting at lower calcium concentrations could be much slower than those reported (70-150 msec) from measurements starting at a high calcium concentration. The *in vivo* course of the GCaMP signal will also be affected by the amount of protein expression because of buffering effects^22^.

We presume that all of these factors play a role in the difference in the respiration modulation seen between ArcLight and the GCaMPs, an idea supported by the result showing that GCaMP6f is somewhat faster than GCaMP3 both *in vivo* and in solution (Figs 4 and 7). While ArcLight’s signals were substantially faster than those of either GCaMP, they were also substantially smaller. Thus, ArcLight and GCaMPs are complementary tools whose use depends on whether the more precise temporal resolution and membrane potential sensitivity of ArcLight outweighs the benefit of improved signal-to-noise ratios and calcium sensitivity of the GCaMPs.

That said, faster sensors will be important in determining brain function. The importance of fast temporal processing in olfactory processing has been emphasized in many recent studies^23^,^24^,^37^. Protein voltage sensors may also be useful in distinguishing the different responses of individual cell types. In the case of olfactory receptor neurons, calcium measurements from their glomerular axon terminals primarily reflect the action potential activity of those cells^7^. However, the apical dendrites of mitral cells have calcium transients that reflect subthreshold membrane potential changes in addition to those caused by action potentials^21^. Thus, calcium measurements from mitral cell apical dendrites likely reflect a combination of subthreshold and action potential activity^20^,^38^. A distinction between these two kinds of activity could be measured using two different protein voltage sensors that are preferentially tuned to different depolarization ranges^39^.

Earlier protein voltage sensors had limited *in vivo* utility in the mammalian nervous system because of poor quantum efficiency, small signal size, slow temporal dynamics and / or poor expression^11^,^16^,^17^,^40^,^41^,^42^. As a result, other attempts to use protein voltage sensors *in vivo* needed extensive averaging and/or processing to reveal stimulus-evoked signals (10 trials^16^, 20 trials^17^, 100 trials^43^, 10 trials^44^, 400 trials^45^, 6-30 trials^46^).

Microbial rhodopsin based sensors (Arch/Arch D95N) have promising *in vitro* performance using high intensity illumination^41^,^47^. However, *in vitro* action potential responses could not be detected using the same imaging apparatus that was used for ArcLight^48^, likely because Arch and Arch D95N have 50-100 times lower quantum efficiency. Because of the requirement for high intensity illumination, those microbial rhodopsin sensors, and later variants^47^,^49^ may not be useful for *in vivo* imaging in the mammalian brain.

Recently published sensors (e.g. MacQ-mCitrine and ASAP1) are reported to have *in vitro* performance comparable to ArcLight, and thus may be promising alternatives^50^,^51^. However, a comparison of *in vivo* performance is not yet possible as there are presently no reports of ASAP1 measurements *in vivo*, and those of MacQ-mCitrine were carried out in a different preparation.

The results from repeated imaging trials were relatively consistent (Fig. 2d), which suggested that substantial photo-bleaching or photodynamic effects were not present with ArcLight or the GCaMPs at the incident intensities we used. Our comparison of intrinsic activity from injected and control hemispheres did not detect a pharmacological effect of either probe on normal bulb function (Fig. 3a-b, d). However, continued attention to these possibilities is desirable. The ArcLight signals were sufficiently large and fast to be distinguished from the intrinsic light scattering and flavoprotein autofluorescence (Fig. 3a-b)^1^,^52^. The autofluorescence signal was very small in the presence of the increased fluorescence from ArcLight.

Our ArcLight and GCaMP AAV1 vectors used different promoters, the choice of which can alter the targeting to- and within neuronal populations. However, both CAG and the human synapsin 1 promoter are known to be highly specific for neurons^53^,^54^, and our histological examination (Fig. 1) suggested that both vectors had similar expression mainly targeted to mitral/tufted cells in the outer layers of the olfactory bulb. Therefore, we don’t believe the promoter choice had a substantial effect on the results presented here.

Anesthesia can have an effect on the response properties of individual mitral cells^29^,^55^, and that of the glomerular population signal^20^. That said, local field potential dynamics are mostly unaffected by the anesthetic used in our study, although they can cause a decrease in the overall power of the signal, and shift in gamma oscillations towards higher frequencies^56^. It is important for future studies to address the role of anesthetic on the glomerular voltage signal.

Our results show that ArcLight is useful for examining population neural activity in the *in vivo* mammalian nervous system, a result that is not predictable from measurements on *in vitro* or non-mammalian preparations. While we hope for the future development of improved voltage sensors (faster and larger signals, responses tuned to specific depolarization ranges, targeting to sub-cellular regions), ArcLight already has sufficient signal size and temporal dynamics to be useful for measuring electrical activity. In cells with a substantial post-synaptic calcium signal, a voltage sensor, can, in principle, be better than a calcium sensor at distinguishing action potentials from subthreshold events. A compelling reason for using a protein voltage sensor over traditional organic voltage sensitive dyes is the ability to target them to specific cell types. The olfactory bulb has a strongly differentiated laminar structure amenable to genetic targeting^20^. While our AAV1 vector appeared to be selective for certain cell populations, this specificity is likely due to a combination of the AAV serotype and promoter. In the future we hope to develop cre-dependent AAV1 vectors that could be used to record from specific cell types, and extend the present 2-photon imaging results towards the goal of single cell optical electrophysiology *in vivo* in the mammalian brain.

## Methods

### AAV Vector

ArcLight A242-2A-nls-mCherry was constructed by fusing ArcLight A242^8^ with the self cleaving 2A peptide sequence followed by nuclear localized mCherry^57^. ArcLight A242-2A-nls-mCherry was inserted into the HindIII site of aavCAG Jx vector (Gene bank JN898959) in a forward orientation to create ArcLight A242-2A-nls-mCherry aavCAG Jx (Fig. 1a). This allowed constitutive expression of the voltage sensor under the CAG promoter in wild type (C57BL/6) mice. An adeno-associated virus serotype 1 (AAV1) of the ArcLight construct was produced at the Penn Vector Core at the University of Pennsylvania (www.med.upenn.edu), and has been made available for distribution. For experiments with GCaMP3 and GCaMP6f, we used AAV1s expressing the gene under the human synapsin 1 (Fig. 1b) promoter (#AV-1-PV1627 and AV-1-PV2822 purchased from the Penn Vector Core).

### Surgery and imaging in adult mice

All experiments were performed in accordance with relevant guidelines and regulations. C57BL/6 mice (JAX, Bar Harbor, MA) were housed and handled, and all experiments were performed in accordance with a protocol approved by the Institutional Animal Care and Use Committees at Yale University and the Marine Biological Laboratory. For all surgical procedures, female mice were anesthetized with a mixture of ketamine (90 mg kg^−1^) and xylazine (10 mg kg^−1^). Anesthesia was supplemented as needed to maintain areflexia, and anesthetic depth was monitored periodically via the pedal reflex. Animal body temperature was maintained at approximately 37.5 C° using a heating pad placed underneath the animal. Local anesthetic (1% bupivacaine, McKesson Medical) was applied to all incisions. Respiration was recorded with a piezoelectric sensor.

For viral injections animals were anesthetized, and a small hole (< 1 mm) was made in the skull directly above the anterior part of one or both olfactory bulbs. AAV1 expressing either ArcLight, GCaMP3, or GCaMP6f was injected using a glass capillary (tip diameter 8–15 μm) approximately 500 μm below the surface of the bulb (Fig. 1c). Virus titres for ArcLight, GCaMP3 and GCaMP6f were 4.6e12 genome copies (GC) ml^−1^, 4.7e13 GC ml^−1^, and 4.3e13 GC ml^−1^, respectively. AAV injections ranged from 0.2 to 2 μl. The preparations in Fig. 1 received 2 μl injections of ArcLight and GCaMP3. The preparations in Fig. 2 received 1.5 (a-c) and 2 μl (d,f-g) injection of ArcLight. The preparations in Fig. 4 received 1.5 μl injections of ArcLight and GCaMP3 and 1 μl injections of ArcLight and GCaMP6f. Injected mice received water supplemented with Carprofen (rimadyl) for a minimum of 3 days. We allowed a minimum of 10 days for sensor expression prior to optical measurements. Mice received either a single injection of ArcLight (16 preparations) or GCaMP3 (1 preparation), injections of both ArcLight and GCaMP3 into separate hemispheres (11 preparations), or both ArcLight and GCaMP6f into separate hemispheres (5 preparations). All of the wide-field fluorescence data are from dual injected preparations except for the results in Figs. 2, 3. The results from single and dual injected preparations were consistent with each other.

For *in vivo* 2-photon imaging using ArcLight, mice were anesthetized and ~ 2 mm craniotomies were performed near the sites of the virus injection over the dorsal olfactory bulbs. The skull and dura were removed and replaced with agarose and a cover glass window. 2-photon imaging was performed with a modified MOM two-photon laser-scanning microscope (Sutter Instruments). Two-photon laser illumination was provided by a mode-locked Ti-sapphire laser (Chameleon Vision, Coherent) and laser scanning was performed with a Cambridge Technology XY galvanometric scanning system (Cambridge Technologies). The tissue was illuminated with 920 nm laser light and scans were performed at varying speeds ranging from frame rates of 3 Hz (512 × 512 pixels) to 100 Hz (512 × 16 pixels). 2-photon optical signals were collected with either a Nikon CFI APO LWD 25 × 1.10 N.A. or a Nikon CFI LWD 16 × 0.80 N.A. water immersion objective (Nikon Instruments), passed through a 510 / 84 bandpass filter (Semrock) and converted to electrical signals with a GaAsP photomultiplier (Hamamatsu H10770).

For wide-field fluorescent imaging, mice were anesthetized, and the bone above both olfactory bulbs was either thinned or removed. The exposure was covered with agarose and sealed with a glass coverslip. The dorsal surface of both hemispheres was illuminated with 485 ± 25 nm light using epifluoresence illumination on a custom antediluvian Leitz Ortholux II microscope with either a tungsten halogen lamp or a 150 W Xenon arc lamp (Opti Quip) and a 515 nm long-pass dichroic mirror. Fluorescence emission above 530 nm was recorded with a sanguine NeuroCCD-SM256 camera with 2 × 2 binning at 125 Hz using NeuroPlex software (RedShirtImaging, Decatur, GA).

For intrinsic imaging, the dorsal surface was illuminated with 485 nm and 530 long-pass emission (autofluorescence; black trace) or 705 nm light (intrinsic reflectance) (Fig. 3a-b). Wide-field optical signals were measured using either a 4 × 0.16 NA (3.5 × 3.5 mm field of view), a 3.5x, 0.1 NA objective (4 × 4 mm field of view), or a 10x, 0.4 NA objective (1.4 × 1.4 mm field of view). All of the presented traces were collected using the 10x (Fig. 2d) or the 4x objective (all other traces); the data in Fig. 4e-h and Fig. 5e were collected with either the 4x or 3.5x objective.

Bath application of TTX was performed in four preparations using a previously described protocol^58^. 4−6 weeks after virus injection, the preparation was prepared as described above except that the dura was also removed. Following an imaging session lasting 1−2 hours, the coverslip and agarose was removed, and a piece of gelfoam (Henry Schein, Melville, NY) soaked with 30-50 μM TTX (Biotium, Hayward, CA) was placed on the brain surface. The gelfoam was left in place for 30 minutes, after which the brain was rinsed with saline and the agarose and coverslip were reapplied.

### Odorant stimuli and delivery

Odorants (Sigma-Aldrich) were diluted from saturated vapor with cleaned air using a flow dilution olfactometer described previously^59^. The olfactometer was designed to provide a constant flow of air blown over the nares. Through a separate set of tubing, odorants were constantly injected into the olfactometer, but sucked away via a vacuum that was switched off during odorant presentation. Cross contamination was avoided by using separate Teflon tubing for each odorant. Odorants were delivered at different concentrations (between 0.5-10% of saturated vapor), although 10% of saturated vapor evoked the most consistent responses, and was used for all the wide-field imaging data presented in the paper. For wide-field imaging experiments odorants were also presented at different durations to examine the response to differing numbers of odorant inhalations. In most of these experiments we tested 1-3 odorants including ethyl tiglate, isoamyl acetate, eugenol, and octanal. All of the traces shown in the figures were elicited by the presentation of ethyl tiglate. The data in the bar graphs in Fig. 4e-h,5e also include responses to isoamyl acetate.

### Data Analysis

Odorant-evoked signals were collected in consecutive (3-6) odorant presentations separated by a minimum of 30 s. The individual trials were manually inspected, and occasional trials with obvious mechanical artifact were discarded. The onset of inhalation was defined as the first downward deflection following the first peak in the respiration recording after the start of the odorant presentation. Response latency values are given relative to the completion of the inhalation. Recordings were either examined as individual trials, or averaged after an alignment procedure where the time of the first inspiration following the odorant presentation was identified and the individual recordings synchronized to this time. Aligned averaged traces were used in Figs 2b,f-g,3a-b,4e-h,5e and 6c-f. All other traces are the result of single trials. Unfiltered traces are shown in Figs 2c,d and 5a (thin red traces). All other traces were Gaussian low pass filtered at 4 or 6 Hz (Figs. 2f,3a,4a,c,5a-d and 6a-b) or at 2 Hz (Fig. 2f,3b). ArcLight signals representing depolarization are shown as upward signals^8^. For the GCaMPs, calcium increases are shown upward. Spatial maps of odorant evoked activation (Figs. 2b,6f) were made by subtracting the temporal average of the 1-2 s preceding the stimulus from a temporal average at the peak (Fig. 2b) or at different time points (Fig. 6f) during the response using frame subtraction in NeuroPlex. The frame subtraction map in Fig. 2b has no spatial filtering. The map in Fig. 6f was spatially smoothed using a low pass 3 × 3 pixel kernel with the center pixel given a weight of 3, and a high pass 41 × 41 pixel Gaussian kernel. Glomeruli were visually identified as circular peaks of activation ~50–100 μm in diameter. The traces in all figures are the averaged signal from pixels in such glomerular sized regions of interest.

Odor-evoked intrinsic optical signals (reflectance) were measured at 705 nm as a test to determine whether the ArcLight and GCaMP3 viral injections had an effect on the odorant responses of the olfactory bulb. Signals were examined in experiments that received an ArcLight or GCaMP3 injection into one hemisphere, with the other used as a control, although intrinsic signals from dual injected hemispheres were also examined in 5 preparations; total glomeruli: N_ArcLight_: 20, N_GCaMP3_: 25. Between 2-5 of the largest glomerular sized peaks of activation were identified using frame subtractions in NeuroPlex, and were used for the comparison of intrinsic signals in injected and uninjected hemispheres. Intrinsic signals recorded in injected and uninjected hemispheres in the same preparation were not different for single injections of ArcLight (Fig. 3d,4 preparations) or GCaMP3 (not shown, 1 preparation). Because we carried out only a single GCaMP3 alone measurement, we also compared the intrinsic signals from dual injections of ArcLight and GCaMP3. The intrinsic signals were similar in both hemispheres (not shown). These data suggested that neither ArcLight nor GCaMP3 injections had a substantial effect on olfactory bulb physiology.

In some experiments with large (> 1.5 μl) GCaMP virus injections a small signal originating from GCaMP3 or GCaMP6f could be detected in the hemisphere contralateral to the injection site. We found that lowering the virus injection volume helped to eliminate this cross-talk. Only preparations without cross-talk were used for the results reported here.

The response latency analysis in Fig. 4e-g was performed in identified glomeruli in trials in which the mouse took only a single sniff of odorant. A single GCaMP3 glomerulus was omitted for the decay analysis due to an exceptionally slow return to baseline. The breath coupling analysis in Fig. 5e was quantified by importing the respiration trace, and the ArcLight, GCaMP3 and GCaMP6f measurements into MATLAB (Fig. 5e). All traces were low pass filtered at 20 Hz and normalized to the maximum optical signal size (0-1). The peak power of the respiration trace was identified between 2-5 Hz for each preparation (ArcLight and GCaMP3 injected: 3.6 ± 0.1 Hz; 6 preparations; ArcLight and GCaMP6f injected: 3.1 ± 0.3 Hz; 5 preparations). A Fast Fourier transform (FFT) was computed on the traces (with a Hanning window) for the 2 s time period before, and during the odorant presentation. The power at that preparations respiration frequency (± 0.25 Hz) was measured for each trace. A repeated measures ANOVA was performed to compare differences in power between sensor type and stimulation time (i.e., before or during odor presentation). After confirming a significant difference in stimulation and sensor, a one-way ANOVA was performed on the power measurements before and during odor presentation to examine significant differences between the three sensors (Fig. 5e, bottom). This analysis was also performed on a subset of glomeruli taken from the most caudal-lateral bulb in each preparation (Fig. 5e, top, repeated measures ANOVA for stimulus: F(1, 48) = 264.5, p < 0.0001, one-way ANOVA before: F(2, 48) = 155, p < 0.0001 and during odor (F(2, 48) = 117.2, p < 0.0001, Tukey tests confirmed ArcLight was largest).

Comparison of signal power at the respiration frequency before and after TTX application were performed by taking the optical signal from a single location on the bulb in each preparation. 1 and 2-sample t-tests were performed to confirm that there were significant decreases in the optical power at the respiratory frequency following TTX application.

The GCaMP3 and GCaMP6f response to repeated inhalations was quantified by measuring the signal size (Δ*F/F*) of GCaMP3 and GCaMP6f at the peak response following each sniff, and normalizing the maximum response to 1. A single region of interest was selected for each preparation as being the most strongly breath coupled glomerular sized region of interest. Paired t-tests were used to compare the normalized GCaMP3 and GCaMP6f signals for the first 3 sniffs. The GCaMP3 and GCaMP6f responses were measured in 8 and 4 preparations, respectively.

Statistical analyses were performed in MATLAB, Origin 9 (OriginLab Corporation, Northampton, MA), SPSS (IBM Corporation, Armonk, NY) or Microsoft Excel.

### Kinetic analysis of ArcLight and GCaMP signals

The onset and decay time constants for the binding of calcium to GCaMP3 and GCaMP6f for Fig. 7 were taken from published values and personal communication^26^,^33^,^34^ (D. Kim, L. Looger, S. Wang, personal communication). The effect of filtering the ArcLight signals with different time constants in Fig. 7f was done using the following resistor-capacitor (RC) low-pass filter:
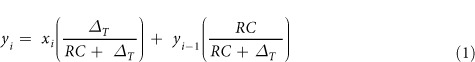
ArcLight kinetics were experimentally measured in HEK293 cells (Fig. 7a,c). Recordings were made in a whole-cell configuration using a Patch Clamp PC-505B amplifier (Warner Instruments). Cells were held at −70 mV, and depolarized to either −30 mV, +10 mV or +50 mV. Optical traces were recorded using wide-field imaging on a Nikon Eclipse E6000FN upright microscope (Nikon Instruments) with a water immersion objective (60 x /1.00 NA), and the fluorescence changes were recorded with a NeuroCCD-SM camera (RedShirtImaging) at 1000 Hz. Probe dynamics were based on the fit to a single exponential equation. Double exponential fits were also calculated but the fit was only slightly improved^8^.

Conversions from time constant to cutoff frequency (Fig. 7a-b) were performed using the equation:
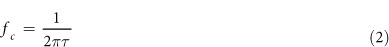


### Histological Methods

After each imaging experiment, mice were given an overdose of euthasol, decapitated, and their brains were dissected and left in 4% paraformaldehyde for a minimum of 3 days. Each olfactory bulb was embedded in 3% agarose, and cut on a vibratome in 50–100 μm thick coronal sections. Mounted sections were coverslipped with VECTASHIELD Hard Set Mounting Medium with DAPI (Vector Labs, H-1500). Slides were examined using either a widefield epifluoresence microscope, or a Zeiss LSM-780 confocal microscope (Carl Zeiss Microsystems). The fluorescence filter sets for ArcLight/GCaMP3, mCherry and DAPI were 480/30 (center wavelength/band width; excitation), LP505 (dichroic) and 535/40 (emission); 540/25, 565LP and 605/55; and 350/50, 400LP and 460/60, respectively. Images were analyzed and figures prepared using Zen Lite 2011 (Carl Zeiss Microsystems) and Adobe Photoshop and Illustrator (Adobe Systems Inc.).

ArcLight, GCaMP3, and GCaMP6f all exhibited strong fluorescence that was detectable without additional amplification steps. Their expression patterns were assessed relative to the cytoarchitecture identified using DAPI fluorescence. Expression patterns were generally consistent with the sections presented in Fig. 1d-k), although there was some variability in the spread of expression from the injection site and in the number of labeled neurons. There was never evidence of injections into one hemisphere labeling cell bodies in the superficial layers of the contralateral hemisphere. However, fluorescence was sometimes present in the deepest layers of the contralateral hemisphere (not shown).

## Author Contributions

D.A.S. and L.B.C. conceived the experiments, wrote the manuscript and created the figures. D.A.S., O.R.B., L.J., and L.B.C. performed the experiments. U.S. created the plasmid for the AAV1 vector. All authors reviewed the manuscript.

## Additional Information

**How to cite this article**: Storace, D. A. *et al.* Monitoring Brain Activity with Protein Voltage and Calcium Sensors. *Sci. Rep.* **5**, 10212; doi: 10.1038/srep10212 (2015).
